# 85 A Machine Learning Model for Estimating Burn Outcome: Analyzing American Burn Association National Burn Repository

**DOI:** 10.1093/jbcr/iraf019.085

**Published:** 2025-04-01

**Authors:** Mariana gutierrez Salazar, Anthony Papp

**Affiliations:** Vancouver General Hospital Burn Unit; Vancouver General Hospital Burn Unit

## Abstract

**Introduction:**

Burn injuries present significant health challenges with serious physical, psychological, and economic consequences. Clinical prediction tools like the Abbreviated Burn Severity Index (ABSI), Baux Score, and Ryan Score are widely used to predict outcomes such as mortality by analyzing variables like age, burn size, and depth. A recent study using a provincial burn registry identified the Baux score as the best predictor of mortality. This study aims to consolidate data on predictors of mortality in burn patients using the American Burn Association National Burn Repository and validate these findings against a provincial burn registry. A secondary goal is to develop enhanced models for mortality prediction to improve clinical decision-making.

**Methods:**

This retrospective cohort study used data from burn patients recorded in the ABA-NBR (2009-2018) and a provincial burn registry (1973-2017). Inclusion criteria required complete data on age, gender, total body surface area (TBSA), burn depth, and inhalation injury. Using R statistical software, multivariate regression and machine learning models were developed to predict mortality and compared with Baux, Revised Baux, and ABSI scores.

**Results:**

Analysis of over 280,000 ABA-NBR patients found age and TBSA to be the strongest individual predictors of mortality. The Baux score remained the most accurate mortality predictor, outperforming the Revised Baux and ABSI (p< 0.001) in this cohort. A machine-learning-based model using sex, age, total TBSA, burn etiology, and inhalational injury demonstrated better accuracy of 96.7% (AUC 0.950; sensitivity 0.700; specificity 0.970) than Baux score which had an accuracy of 94.9% (AUC 0.929; sensitivity 0.351, and specificity 0.997) in this cohort.

**Conclusions:**

The Baux score is proven in this study continue to be a good and easy-to-use predictor of mortality in burn patients. While the new machine-learning model showed improved accuracy, its complexity may limit its use in clinical practice. Future work is aimed at expanding candidate variables using the ABA-NBR to determine if there are other key variables that may be important for prediction of mortality. Finally, a provincial burn registry will be used for final validation of this tool’s applicability in diverse populations.

**Applicability of Research to Practice:**

The presented work provides further validation of the Baux score and encourages its ongoing application in the prediction of burn associated mortality.

**Funding for the Study:**

Internal Educational Funding

